# Bad blood: inequity in hemophilia care

**DOI:** 10.1016/j.rpth.2023.102290

**Published:** 2023-12-07

**Authors:** Lauren E. Merz, Angela C. Weyand

**Affiliations:** 1 Department of Medical Oncology, Dana-Farber Cancer Institute, Boston, Massachusetts, USA; 2 Division of Hematology, Department of Medicine, Mass General Brigham, Boston, Massachusetts, USA; 3 Department of Pediatrics, University of Michigan Medical School, Ann Arbor, Michigan, USA

The United States has a long and complicated history with race and ethnicity. Skin color and family origin have been weaponized to maintain control for a few, and this has resulted in systemic racism that has been melded into the fabric of our society. Systemic racism is defined as embedded structures or entrenched practices that perpetuate unfair treatment or impose barriers to opportunities [1]. Examples of this include redlining, which excluded people of color from accessing high-value property and building generational wealth. This can subsequently lead to food deserts, low-performing schools, and higher crime rates- perpetuating a vicious cycle of poverty and poor health [1]. It is important to differentiate systemic racism from race. Race is a social construct based on physical appearance, social factors, and cultural background [2]. At times, race has been used as a proxy for genetics within medicine. However, we now know that there can be more genetic variation within people of the same racial group than between those of different racial groups [2]. Thus, it is critical to acknowledge that racism is the risk factor—not race.

The concept of race and systemic racism is especially important to recognize in the field of medicine. Medicine has been both susceptible to and culpable of the enmeshment of race and racism into daily practice. In some instances, the inclusion of race into medicine had malicious intent, such as the plantation physician Samuel Cartwright, who published on a 20% deficiency in the lung capacity of enslaved Black Americans to medically demonstrate inferiority and justify forced labor [3]. In other cases, the inclusion of race in our assessments of patients may be used as a poor proxy for genetics or as a misguided reflection of socioeconomic factors. Increasingly, there has been a focus on assessment and documentation of racial disparities within medicine. While this has predominantly arisen from good intentions, we must be cognizant of the way we discuss race. We must recognize that race is an inappropriate proxy for other factors such as genetics, disease biology, or systemic racism.

We see the assessment and documentation of race, ethnicity, and disparities in hemophilia care as well. Non-Hispanic Black patients are less likely to receive prophylaxis and less likely to be on home therapy than non-Hispanic White patients [4]. Black and Hispanic patients have a 2-fold higher frequency of inhibitors than White patients despite having similar genetic mutations [5]. One study found that immune tolerance induction (ITI) is less likely to be successful in Black patients [6]. Despite similar levels of adherence to treatment, non-White patients report higher levels of chronic pain and lower quality of life [7]. Black patients have worse functional status than White patients across all age groups, regardless of inhibitor status [8]. Across every metric that has been studied, non-White and especially Black patients have worse outcomes than White patients. However, this is due to systemic inequity—not biology.

The paper “Association of Race/Ethnicity with Receipt of Immune Tolerance Induction in Severe Hemophilia A in the United States” by Kempton et al. [9] further explores racial and socioeconomic factors associated with ITI for inhibitor eradication among persons with hemophilia A complicated by an inhibitor. White patients were more likely to travel farther to their treatment center compared to those of other races or ethnicities. However, access issues like driving distance and travel time were not significantly associated with ITI treatment. There was no significant difference by race or ethnicity in receiving care at high- vs low-volume centers. In unadjusted models, there was no significant difference in ITI treatment by race or ethnicity. However, in models accounting for other clinical and access factors, Black (adjusted prevalence ratio, 0.91; 95% CI, 0.84-0.99) and Hispanic (adjusted prevalence ratio, 0.84; 95% CI, 0.75-0.93) patients were less likely to receive ITI treatment compared to White patients.

This paper found lower rates of ITI among Black and Hispanic patients in adjusted models compared to White persons with hemophilia A complicated by an inhibitor. Race or ethnicity is not the risk factor and likely acts as a proxy for other barriers. Likely contributors to the observed disparities in hemophilia care and outcomes are outlined in the Figure. For example, a previous study showed that ITI treatment is less frequently successful in Black patients, which may implicitly or explicitly influence decisions around offering ITI treatment [6]. Historically, lack of patient trust in the healthcare system has been invoked as an explanation for racial disparities. However, we believe that providers should reflect on their own trustworthiness rather than shift blame to patients. Other explanations for the disparities observed by race and ethnicity include sociodemographic differences such as barriers from cost, transportation, insurance, sick leave, or language concordance. Interestingly, the authors found no difference in ITI treatment by distance or travel time, making this an unlikely explanation for the disparity observed by race. Unfortunately, assessment of most of these other factors was outside of the scope of this study. Future studies must assess the underlying causes that drive the difference in ITI by race and not rely on race alone.

**Figure 1:**
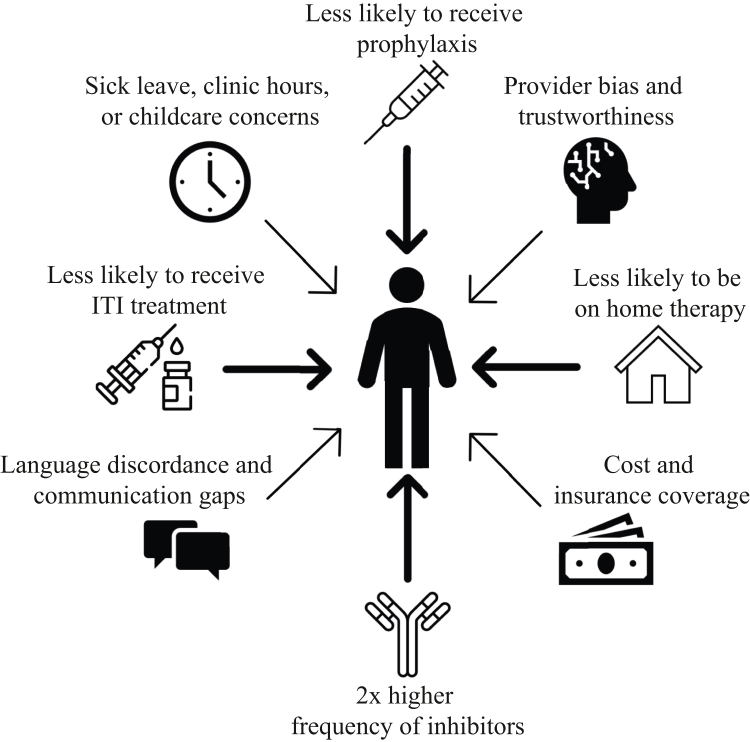
Contributors to racial and ethnic disparities in hemophilia care and outcomes.

In the meanwhile, this study is a call to action that providers must recognize that there are disparities by race in hemophilia treatment, and radical change is necessary. This requires reparative justice—a form of justice that centers on those who have been harmed, repairs past harms, stops present harm, and prevents the reproduction of harm [1]. The volume of work to be done can be overwhelming, but addressing even a small aspect of a single issue is meaningful and worthwhile. We must not fall into apathy. Engaging with individuals and the community who have been impacted to understand needs and barriers is essential. The first step involves educating yourself as much as possible on the historical and current issues in the impacted community as well as the relationship of the community with the healthcare system. Asking patients for their perspective can be illuminating. Patients may be open to sharing their experience, but it is important to remember that it is not a minoritized patient’s role to educate others on their experience and they are not spokespersons for the entire community. Additionally, it is important to ensure that there is diverse racial and ethnic representation and inclusion in hemophilia organizations. This can help with bidirectional knowledge exchange between providers and minoritized communities around patient concerns, development of new initiatives, and awareness of new therapies. We must strive to become worthy of the trust of our patients by building relationships, demonstrating sustained interest and care, and honoring autonomy.

In addition to engaging with minoritized populations, providers must actively interrogate their implicit biases and put processes in place to overcome deep-rooted assumptions and behaviors. The Harvard Implicit Association Test is an eye-opening tool to understand our own hidden implicit biases. Acknowledging and confronting our unconscious biases is the first step to overcoming them. We also recommend implementing protocols or checklists for care whenever possible. Systems like this have been shown to reduce the influence of implicit biases. For example, a hemophilia center could include a treatment best practices section in each patient’s chart and explicitly record the last time ITI was offered and the rationale for why ITI has not been completed to date. The process could prompt critical, objective evaluation, which can help overcome deep-rooted assumptions. Larger, long-term goals include changes to our healthcare systems, such as lowering barriers to hemophilia treatment by expanding clinic or infusion room hours, providing childcare or transportation stipends, and advocating for adequate insurance coverage for standard-of-care treatments. Identifying racial disparities in medicine is a necessary first step, but we must simultaneously emphasize that race is not the risk factor. Rather, we must find and fix the true root of the disparity to make lasting and meaningful change.
